# Porosity and Pore Size Distribution of Native and Delignified Beech Wood Determined by Mercury Intrusion Porosimetry

**DOI:** 10.3390/ma12030416

**Published:** 2019-01-29

**Authors:** Selin Vitas, Jana S. Segmehl, Ingo Burgert, Etienne Cabane

**Affiliations:** 1 Wood Materials Science, ETH Zürich, Stefano-Franscini-Platz 3, CH-8093 Zürich, Switzerland; svitas@ethz.ch (S.V.); jana@segmehl-energie.de (J.S.S.); iburgert@ethz.ch (I.B.); 2 Applied Wood Materials, EMPA—Swiss Federal Laboratories for Materials Science and Technology, Überlandstrasse 129, CH-8600 Dübendorf, Switzerland

**Keywords:** mercury intrusion porosimetry, delignification, wood, freeze-drying

## Abstract

The complex hierarchical structures of biological materials in combination with outstanding property profiles are great sources of inspiration for material scientists. Based on these characteristic features, the structure of wood has been increasingly exploited to fabricate novel hierarchical and functional materials. With delignification treatments, the density and chemistry of wood can be altered, resulting in hierarchical cellulose scaffolds with enhanced porosity for the fabrication of novel hybrid materials. In the present study, focusing on acidic delignification of beech wood and its influence on porosity, we report on a structural characterization and qualitative assessment of the cellulose scaffolds using mercury intrusion porosimetry (MIP). To account for the effect of water removal from the hygroscopic structure, different drying methods—e.g., standard oven and freeze-drying—were applied. While native beech wood is characterized by the presence of macro, meso and micro pores, delignification altered the porosity, increasing the importance of the macropores in the pore size distribution. Furthermore, we showed that the final porosity obtained in the material is strongly dependent on the applied drying process. Samples delignified under harsh conditions at high temperature (mass loss of ~35%) show a 13% higher porosity after freeze-drying compared to oven-dried samples. The obtained results contribute to a better understanding of the impact of the delignification and drying processes on the porosity of cellulose scaffolds, which is of high relevance for subsequent modification and functionalization treatments.

## 1. Introduction

The need to replace oil-based materials by sustainable alternatives has raised the interest for plant-based alternatives. In this regard, wood and wood-based materials have gained a lot of interest in the material science community [1,2,3,4]. In addition, the complex hierarchical structures present in biological materials have been intensively studied, and a number of concepts and design principles inspired by nature could be extracted and successfully implemented for the development of advanced materials [5,6,7,8,9,10,11,12,13,14,15,16]. These concepts have been a source of inspiration for the modification of wood to gain functional renewable materials [17]. The approaches go well beyond the classical wood technology striving for novel applications, as most of them use the anisotropic and porous wood structure as an eligible scaffold for functionalization treatments [1].

Wood cell walls are essentially constituted of three main components: two polysaccharide components, cellulose and hemicelluloses, and lignin, an aromatic crosslinked polymer [18,19]. Lignin and hemicelluloses form the matrix, in which the cellulose fibrils are embedded and lignin is the main component of the middle lamella, which sticks the wood cells together. The development of functional lignocellulosic materials usually requires a chemical modification addressing the wood cell wall biopolymers [17]. In this regard, several research groups came up with a new approach for bulk wood modification, based on the removal of lignin [1,3,12,14,16,20,21]. Originally developed for pulp and paper production to gain individual cellulose fibers [22,23], delignification processes are now reconsidered to remove lignin from wood while retaining its hierarchical structure and fiber directionality, opening up new avenues in materials engineering [21].

In the case of wood, the native structure is worth preserving, and researchers proposed that the removed lignin could be advantageously replaced by a synthetic matrix with tailored properties. In recent works, wood has been made transparent by impregnating the delignified wood with a polymer of similar refractive index [11,24,25,26], opening up applications for wood materials based on new optical properties. In another example, the selective removal of lignin resulted in a flexible wood material, with potential applications in medical devices [27]. In other studies, cellulose-polymer composite materials with unique mechanical properties were fabricated through the densification of previously delignified wood samples [14,28,29].

From a processing point of view, bulk wood modification is challenging despite the porous structure. Transport of reactants to the reaction sites, mainly hydroxyl groups of the cell wall polymers, deep in the macroscopic bulk wood structure and into the cell wall is diffusion driven and possibly hindered by capillary forces [14,30,31]. The partial removal of lignin is believed to increase porosity and open new pathways for the transport of solutions deep inside the material, which should facilitate a subsequent impregnation step for further functionalization [32,33].

One novel potential application of wood is its use as a filter or membrane due to the intrinsic anisotropic porosity of the scaffold [14,30,31]. Modified beech wood has been used as an adsorbent for copper [34]; however, to compete with other membrane or filtration materials, the surface area of wood needs to be increased. Delignification of wood may result in a more porous material with a larger surface area, which could facilitate the interactions between dissolved compounds and functional groups. However, this requires an improved understanding of the delignification process and in particular of its effect on the porosity of the wood structure.

The characterization of both native and delignified wood scaffolds is crucial to establish scalable processes for an efficient wood modification. In particular, the density and the porosity of the materials have to be determined, to provide important knowledge for the processing steps and for the final application. The effects of wood delignification can initially be assessed by microscopic observation; however, alterations in micro- and submicroscale porosity require in-depth characterization. Various methods can be employed, such as stereology, radiation scattering, pycnometry, gas adsorption, mercury intrusion, fluid flow, calorimetry and many others [35]. One should be aware that various methods use different means to provide the same physical quantities and not all of them can be compared: pore sizes determined by microscopy tend to be smaller than with Mercury Intrusion Porosimetry (MIP), and nitrogen adsorption can access smaller pores, and thus show a different size distribution. However, good consistency has been found for the determination of the surface area when comparing nitrogen adsorption and MIP [36,37]. 

One of the advantages of MIP is the possibility to investigate the sample as a whole over a wide range of pore sizes. Hence, various wood species have already been successfully characterized with MIP [38,39]. However, the investigation of delignified wood samples is lacking, especially when considering the drying process. Indeed, drying is an important step when considering porous materials [40], as it greatly influences their structure and material properties. Since wood is a hygroscopic material, bound water is always present within the cell walls when stored under ambient conditions. While drying, this water is removed leading to structural rearrangements. This is due to the gradient in water content resulting in local stress concentrations at the tissue level and the surface tension of the receding water droplets in the cell walls [41]. This pore closure generates stresses within the material, possibly leading to larger cracks [42]. On the contrary, for freeze-drying, sublimation is believed to prevent these effects and retain the voids [12]. 

In this work, we report on the characterization of native and delignified beech wood (*Fagus sylvatica*), which are intended to be used as filtration media for particles larger than 0.1 μm. The objective was to increase the porosity of the material through acidic delignification and show how the pore size distribution is affected by this procedure and subsequent drying steps. The delignification follows a previously reported acidic delignification protocol, which was shown to be more efficient in removing lignin compared to a basic condition process [21]. As reported in previous studies, the selectivity of the delignification is not only based on the method used, but also on the initial chemical composition of the biomass [43]. In case of treating wood with the acidic delignification protocol, an additional removal of hemicelluloses takes place, which has not been further analyzed as the focus of the study is on structural changes.

When compared to other membrane and filter materials, wood is inexpensive and widely available. To maintain this competitive advantage, the applied delignification method should be easily up-scalable with little processing of the final product. For this reason, we investigated straightforward drying methods, i.e. drying in an oven or a freeze-drying process. The effects of the delignification treatment and the drying process on the bulk were characterized by scanning electron microscopy (SEM) to observe the structural changes, and by MIP to determine the porosity in more detail. The pore sizes observable by this method are in the same ranges as natural openings present in wood and in filter media. We observed an alteration of the wood structure resulting from the disassembly of various wood cells (vessels, libriform fibers, fiber tracheids and ray cells). This goes along with an overall increase of porosity (20%) and a clear shift in the distribution of pores towards larger diameters.

## 2. Experimental Section

### 2.1. Materials

Native beech wood samples were cut perpendicular to the fiber direction into thin discs of dimensions Ø 2.5 cm × 1 mm (Ø RT × L). Acetic acid (Sigma-Aldrich Chemie, > 99.8% puriss, Buchs, Switzerland) and hydrogen peroxide (Chemie Brunschwig AG, 35wt.% stab., Basel, Switzerland) were used as received.

### 2.2. Methods

#### 2.2.1. Delignification

The delignification reaction was conducted according to a previously reported method [21]: samples were oven dried (65 °C) to determine their dry weight. Then, they were immersed in a solution of acetic acid and hydrogen peroxide 1:1 (*v*/*v*) for 8 h at 60 °C (“mild” conditions) or 4 h at 80 °C (“harsh” conditions); 10 replicates were used for each condition. After reaction completion, the samples were washed in water for 5 days; the water was exchanged every day. The degree of delignification (DD) was calculated as the weight loss (in percent) of the dry material before and after treatment.

#### 2.2.2. Drying

After reaction, samples were dried under two different conditions: in an oven (OD: oven-dried) at 65 °C for 24 h or freeze-dried (FD) with liquid nitrogen in a freeze-dryer (Alpha 1-2 LDplus, Christ, Osterode am Harz, Germany). The freeze-dried samples were then placed under high vacuum at room temperature for 5 days.

#### 2.2.3. Sample Equilibration Prior to MIP Measurements

The influence of the storing conditions was investigated for the native wood. For the MIP analysis, the samples needed to be dry, as bound water in the pores may result in severe inaccuracies of the measurement. Since wood is a hygroscopic material, it is advised to dry the sample prior to analysis [38,44]. In our study, we compared two different equilibration methods: samples were freeze-dried and kept under vacuum until the measurement or samples were kept under ambient relative humidity (RH = 50%). According to our results (Figure S1), a final freeze-drying of the samples prior to analysis had no additional effect.

#### 2.2.4. Density Determination

In order to determine the density of the samples, their mass was recorded with a balance and the volume was measured using a pycnometer (GeoPyc 1360, MICROMERITICS, Norcross, GA, USA) by displacement of a medium not intruding the pores of the material under these conditions. The medium used for this purpose is called DRYFLO^TM^; it is composed of spherical beads of Teflon and contains graphite as a lubricant. The specific density was determined by using Helium pycnometry (AccuPyc 1330, MICROMERITICS, Norcross, GA, USA). In this case, the displaced medium, helium, was able to fill the smallest pores. The difference in pressure was used to calculate the density. The density directly depended on the weight and the volume of the sample.

#### 2.2.5. Porosity

The porosity (Φ) can be calculated from the bulk density (*ρ*) and the specific density (*ρ_s_*) as follows:

$$\Phi =1-\;\frac{\rho }{\rho_{s}}$$

This porosity accounts for all pores, open and closed. According to practice, pores ranging from 3.5 nm up to 100 μm can be measured through mercury intrusion porosimetry (MIP); the limit of the measurement was determined by the geometry of the sample [36].

The analysis was performed with a Pascal 140 + 440 instrument from POROTEC (Hofheim am Taunus, Germany). The measurement has been carried out according to experimental conditions described in previous work [38,39]. The volume of the pores was determined by the quantity of mercury intruded under a known pressure. From the volume of mercury intruded, the total pore surface area, and hence, the total internal volume, could be calculated.

Mercury is a non-wetting fluid, and therefore cannot penetrate a porous solid through capillary forces. For reasons of simplification, we approximate wood as a bundle of small cylindrical capillaries [45]. Therefore, the penetration of mercury into this porous body can be described by the Washburn equation. It relates the radius of the pores (*r*) with the measured pressure (*p*), the contact angle (*θ*) of mercury and its surface tension (*γ*) being constants:

$$r=\;-\;\frac{2\gamma \;\cos\theta }{p}$$

#### 2.2.6. Electron Microscopy

Radial sections were prepared for microscopy analysis, in order to investigate the lumina of the wood cells. For high resolution images, samples were sputtered with a mixture of 80% Pt and 20% Pd with a thickness of 9 nm. Images were acquired using a low accelerating voltage (10 kV) and a constant working distance (10.0 mm) under high vacuum in a FEI Quanta 200F microscope (Hillsboro, OR, USA).

## 3. Results and Discussion

### 3.1. Delignification of Wood

**Figure 1:**
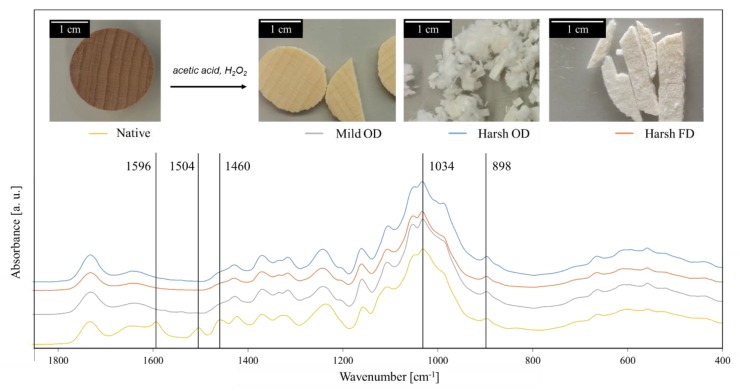
Delignification of beech wood samples under mild and harsh conditions, oven dried (OD) or freeze-drying (FD), and the corresponding Fourier-Transformed Infra-Red (FTIR) spectra with characteristic vibrations for lignin (1596, 1504 and 1460 cm^−1^) and cellulose (1034 and 898 cm^−1^).

The degree of delignification (DD, in percent) can be calculated from the weight loss of the sample after the delignification procedure. The higher temperature process (“harsh” delignification) led to greater weight loss (DD = 34.91 ± 0.01%) than the “mild” delignification conducted at lower temperature (DD = 27.55 ± 0.01%). Beech wood is composed of about 20% lignin, 45% of cellulose and 35% hemicellulose [19,46]; the extractives account for about 1% of the weight [19]. Hence, when the samples have lost more than 20% of their weight, in addition to lignin, hemicelluloses and extractives have also been removed, at least partially, from the structure. Since lignin located in the middle lamella contributes to the binding between wood cells of various types [19,25,46] and is also a main component of the cell wall matrix, it contributes together with hemicelluloses to the mechanical integrity of the wood structure [47]. Hence, their substantial removal results in a loss of mechanical stability, which has been observed for our samples: no noticeable difference in mechanical stability can be seen after a “mild” delignification in comparison to the control group, whereas samples delignified under “harsh” conditions were easily broken and exhibited a fluffy and fragile structure. Additionally, the delignification resulted in the discoloration of wood [12,21,27]. This was caused by the changes in the chemical environment of some chromophoric centers and the complete removal of other ones [48]. As shown in Figure 1, discoloration of the sample increased proportionally to lignin removal, until a white material was obtained. Higher temperature and longer reaction times will completely disrupt the material integrity and result in an aqueous suspension of cellulose fibers. In this study, reaction times and temperature of the delignification were optimized in order to preserve the wood scaffold while reaching high weight losses.

Along with the loss of mechanical integrity and the discoloration, the disappearance of vibrations characteristic for lignin (1596, 1504 and 1460 cm^−1^) was observed by FT-IR spectroscopy [43,49], confirming the removal of lignin (Figure 1). The cellulose appears to be only marginally affected by the delignification, as shown by the vibrations characteristic for the cellulose backbone (1034 and 898 cm^−1^) [49].

**Figure 2:**
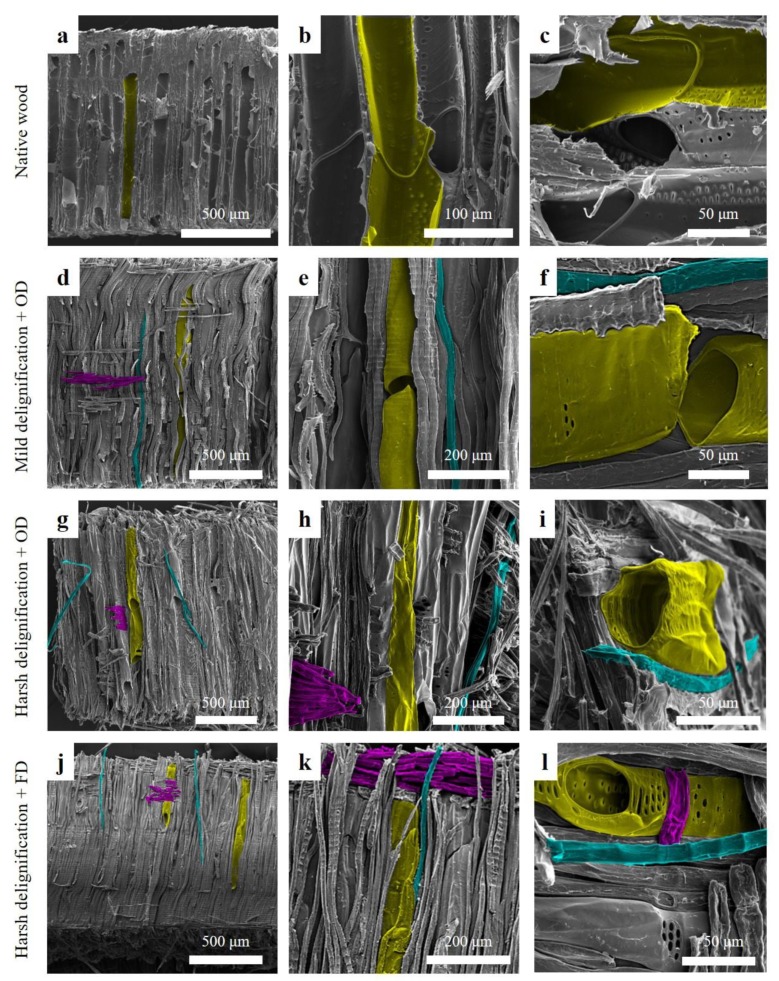
SEM images showing the effect of delignification on the structure of beech wood with increasing removal of lignin and various drying processes. To help visualization, wood anatomical features are highlighted in some images: vessels are inked in yellow, fibers in turquoise and ray cells in violet. (**a**–**c**) Native wood, (**d**–**f**) beech wood after mild delignification and OD; (**g**–**i**) beech wood after harsh delignification and OD; (**j**–**l**) beech wood after harsh delignification and FD.

Changes of the wood on the structural level after delignification can be seen in electron microscopy studies, as shown in Figure 2. The structure of beech wood is constituted of thick walled fibers and thin walled, tube-like vessels aligned in longitudinal direction and ray cells running in the radial direction (Figure S2). The single cells are connected through pits, which allow for the transport of fluid in between the cells and throughout the entire macroscopic structure. Each vessel is composed by several vessel elements aligned end-to-end with the next, which are mostly separated by simple perforation plates visible in Figure 2b,c.

As lignin is partially removed through mild delignification (Figure 2d,f), we observed several structural changes. Fibers became more visible as they detached partially from the bulk (Figure 2e). Vessels were disassembled, since gaps appeared between single vessel elements, as shown in Figure 2f. Overall, the structure kept its integrity; however, as the middle lamella is affected by the treatment, the wood elements started to disassemble. The observations suggested that the amount of voids in the mild delignified samples should increase when compared to native wood. 

The disassembly of the wood cells was further pronounced after harsh delignification (Figure 2g–i). In addition, the wood cells seemed to collapse and the overall structure became more compact (Figure 2g,h). The complete removal of lignin, together with some hemicelluloses and extractives, strongly weakens the cell wall material. Upon drying, the soften cell walls were more likely to collapse, promoting a “compacting” of the entire structure. This could be an indication that the porosity of the harsh delignified samples was lower when compared to the mild delignified samples. 

As shown in Figure 2j–l, in the highly delignified freeze-dried samples, the wood elements are clearly separated from each other, as shown by gaps in between fibers and vessel elements. This resulted from the removal of the middle lamella. However, as opposed to the oven dried samples, the wood individual elements did not seem to collapse; the overall structure was maintained, similar to the mild delignified samples. 

The SEM investigation shows that the delignification resulted in an alteration of the bulk wood structure. We observed the separation of the wood cells—vessels, fibers and ray cells—caused by the removal of the intercellular material. According to our observations, both the reaction conditions and the drying method seem to have an important influence on the porous structure of the delignified samples. 

### 3.2. Porosity Measurements

In order to analyze the results of the MIP, a classification different from the one commonly suggested by IUPAC was chosen [35], based on the work by Plötze et al., who assigned four pore size classes to beech wood: two classes of macropores (pore radii 2–58 μm and 0.5–2 μm), mesopores (80–500 nm) and micropores (3.6–80 nm) [38]. This pore size distribution was also observed in our results, summarized in a histogram given in Figure S1 (values are given in Tables S1–S3). Structural elements and anatomical features were assigned to each of these pore classes. In the native material, vessels represented the macropore contribution, whereas pits and fiber lumina were characteristic for the smaller category of macropores. Additionally, smaller macropores typically corresponded to perforation plates, as well as cracks in-between fibers. Smaller pit openings as well as the porosity of the pit membranes could also belong to the mesopores [38,50,51], whereas the voids presented in the cell wall could be assigned to the micropores [39]. 

The amount of mercury intruded at a certain pressure was given by the volume-pressure curves generated during the MIP measurements (see Figure S3). The relative volume of a defined size can be derived from these curves, and the shape of the curve can also provide information about the internal geometry. In our case, the volume-pressure curves indicated the presence of ink-bottle pores corresponding to the spherical model. This was characterized by steep intrusion plots, since high pressure is needed for the mercury to enter the pores through a smaller neck, which led to a large pore being filled up very slowly. The flattening of the extrusion curve was due to the samples retaining mercury [36], which was due to the interconnectivity of the pores within wood [52]. Using tomography and image analysis, the work by Hass et al. has shown that wood vessels are interconnected within each other [53] and such a pore network might be approximated as ink-bottle shaped pores [52].

**Figure 3:**
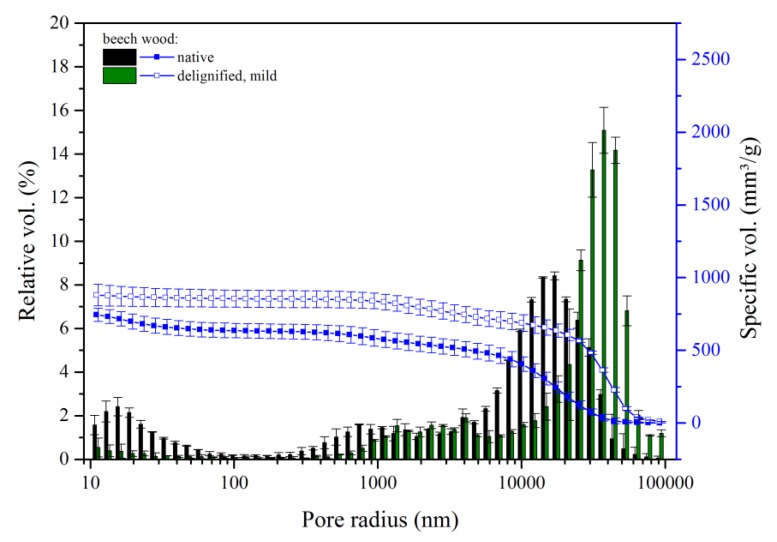
Porosity of native versus mild delignified wood, both oven dried after delignification. Cumulative pore volume and histogram of relative pore volume as a function of the pore radius of native and delignified beech wood following the mild delignification method (60 °C, 8 h).

As shown in Figure 3, for an OD mildly delignified sample, delignification resulted in a significant reduction in the relative volume of the micropores (<80 nm); the distribution of mesopores and the smaller class of macropores (80 nm to 2 µm) was also affected by this treatment: their relative volume decreases and the pore size distribution was shifted to larger radii.

**Figure 4:**
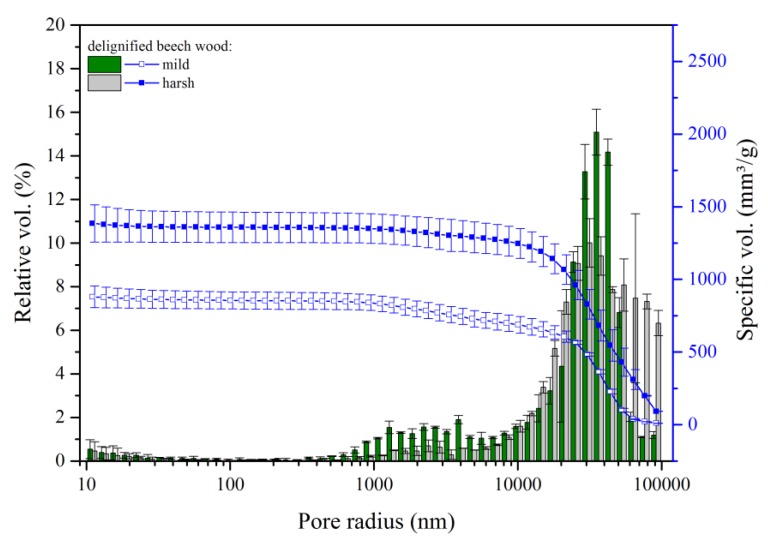
Effect of the extent of delignification on porosity of beech wood. Cumulative pore volume and histogram of relative pore volume as a function of the pore radius of delignified beech wood following the mild (60 °C, 8 h) and the harsh (80 °C, 4 h) delignification method (in both cases, samples were oven dried after delignification).

For delignification at high temperature (DD ≈ 35%), the reduction of the volume of small pores was even more pronounced (Figure 4). Simultaneously, the relative volume of pores with radii larger than 30 µm was increasing. Again, this effect was more prominent for the harsh delignification method. Overall, we observed that delignification generated a shift of the pore size distribution towards higher values, up to 100 µm, see Figure 4. 

These shifts can be assigned to the different features observed in the SEM images: delignification generates gaps (mild delignification) and cracks (harsh delignification) in the wood structure. The voids or pores created during delignification have radii around 20 to 50 μm, according to the porosity measurement, which correlates well with the observations made by SEM (see Figure 2f,i). In addition, very large pores were detected by MIP after high temperature delignification and drying in an oven. This is likely due to the observed delamination and therewith loss in mechanical integrity: bundles of collapsed fibers and vessels still stuck together, but moved or slid against each other, creating large cavities in the bulk of the samples. Such voids accounted for the macropores with radii larger than 50 μm.

**Figure 5:**
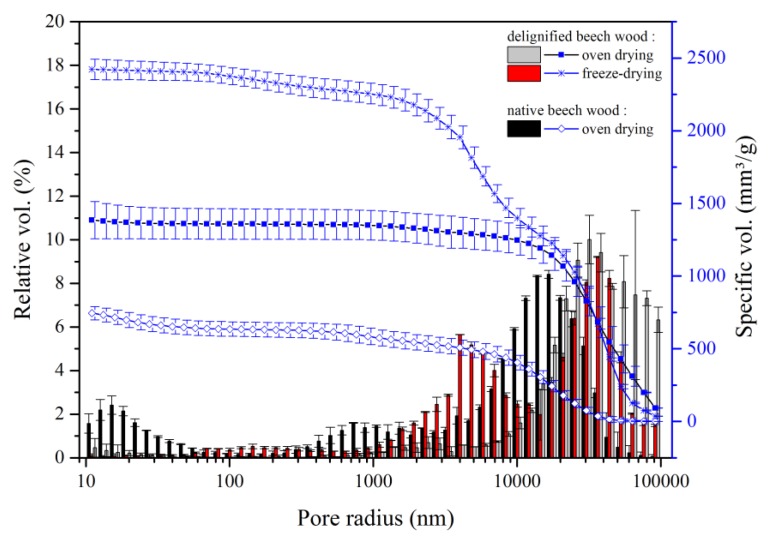
Effect of drying process on porosity of delignified beech wood. Cumulative pore volume and histogram of relative pore volume as a function of the pore radius of native beech, conventionally dried, and delignified wood following the harsh method (80 °C, 4 h) dried by freeze-drying and oven dried.

Figure 5 compares the pore size distribution of delignified samples following the high temperature protocol but with different drying conditions. 

A new pore class in between radii of meso and macropores (0.1–1 µm) is only clearly observed in freeze-dried samples. Both drying methods led to a clear shift towards higher pore sizes. The harsh delignified samples dried in an oven showed one dominant peak in the range of 10–100 µm pore sizes, whereas freeze-dried samples exhibited two peaks centered around 4 and 35 µm in the macropore region.

The spaces originating between the cells due to the removal of the middle lamella have been assigned to the bigger pores, visible in all samples. When considering the freeze-dried samples, a large portion of the void volume was still assigned to smaller pores (distribution centered on 4 µm). Freeze-drying is fast enough to prevent the collapse of the cells and cell walls, preserving some of the native porosity, including some of the mesopores and the lower class of macropores (together from 80 nm to 2 µm). In addition, during freeze-drying, there is no compacting of fibers into bundles leading to large cavities, which minimizes the amount of larger macropores.

### 3.3. Determination of Specific Volume and Area

Removing a constitutional compound should increase the number and size of voids within the material. However, as shown in Figure S4 and summarized in Table S4, mild delignification approaches have led to only little increase in internal volume. Only samples delignified using the harsh treatment displayed larger internal volumes, especially after a freeze-drying step. As the structure of the material is maintained, the loss of about 30% by weight of material necessarily led to an increase in specific volume. The porosity was increased by 13% between native wood (52%) and harshly delignified/oven-dried samples (65%). A combination of harsh delignification and freeze-drying resulted in a sample porosity of 78%, which is again an increase in porosity of 13% compared to the harshly delignified/oven-dried samples. Similarly, the harsh delignification clearly increased the specific surface area (Figure S4). The increase in surface area can be linked with the disassembly of the vessel and fiber network. The more intercellular space was removed, the more surface was uncovered. Freeze-drying prevented the collapsing of the cells; in consequence, the resulting surface area was higher than after drying in an oven. 

## 4. Conclusions

We have shown that the pore size distribution of beech wood is greatly affected by the delignification. The contribution of the micro and mesopores to the relative volume fraction declines with an increasing degree of delignification, accentuating the importance of larger pores (macropores). In addition, the drying process after delignification greatly influences the final porosity. 

Fibers and vessels tend to collapse due to the loss of intracellular material, which is suspected to decrease the variability of the pore sizes. However, it should be possible to preserve the newly formed porosity by avoiding the collapsing of the cells and limiting the formation of bigger cracks within the material. The fast removal of the water by freeze-drying possibly prevented the delignified wood structure to collapse at the macroscale and at the cell wall level, preserving the structural arrangement at different length scales, which resulted in the large specific volumes and specific areas observed. Based on our results, the overall gain in porosity could be controlled by adjusting the delignification conditions. 

MIP has been shown to be a suitable method to measure the porosity of wood with regard to a wood filter application, as it can acquire the size of the measured cavities in a wide size range (3.6–100 μm). The pores could be classified within the various classes commonly used and they could be attributed to structural features visible under the microscope up to the micro scale. While other characterization techniques would be required to complement the here presented findings, the MIP measurements provide a first insight into the structural changes of the wood caused by delignification. These results will be of high interest for further investigating impregnation processes, and for the design of wood filter materials exploiting the hierarchical porous structure of wood. 

## Supplementary Materials

The following are available online at http://www.mdpi.com/1996-1944/12/3/416/s1, Figure S1: Cumulative pore volume and histogram of relative pore volume as a function of the pore radius of beech wood held under ambient conditions at room temperature after oven drying (OD + RT) or with an additional freeze-drying step (OD + FD) prior to measurement. SEM imaging of the cross-section of native beech wood exhibiting various sized cavities in its structure in the range of the (**a**) upper and (**b**) lower level of the macropores; Figure S2: Schematic representation of wood tissue (**a**) and the disassembly of the cells (fibers, tracheids, vessels, ray cells) from the bulk (**b**–**d**) upon the weakening of the intercellular matrix through delignification; Figure S3: Volume-Pressure-Curves showing the intrusion curve (red/purple line) and the extrusion curves for raw (lilac) and delignified wood (grey) and the drying method (OD: oven dried, FD: freeze-dried); Figure S4: Overview of the MIP analysis (pore size distribution, total specific surface area and pore volume) of native and delignified beech wood under mild or harsh conditions, subjected to oven drying (OD) or freeze-drying (FD); Table S1: Pore sizes and pore size distribution of native beech wood from mercury intrusion porosimetry; Table S2: Pore sizes and pore size distribution from mercury intrusion porosimetry of delignified beech wood under harsh conditions; Table S3: Pore sizes and pore size distribution from mercury intrusion porosimetry of delignified beech wood under mild conditions; Table S4: Total pore volume and surface area as well as porosity determined by mercury intrusion porosimetry (MIP) and pycnometry for native and delignified beech wood.
