# Supplementary material for: Porosity and Pore Size Distribution of Native and Delignified Beech Wood Determined by Mercury Intrusion Porosimetry
Source: Materials (Basel). 2019 Jan 29;12(3):416. doi: 10.3390/ma12030416 (PMC6385036; doi:10.3390/ma12030416)
Supplement: Supplementary file 1 [file materials-12-00416-s001.pdf]

Supplementary

Porosity and Pore Size Distribution of Native and Delignified Beech Wood Determined by Mercury Intrusion Porosimetry

Selin Vitas [1,2], Jana S. Segmehl [1,2], Ingo Burgert [1,2] and Etienne Cabane [1,2,*]

[1] Wood Materials Science, ETH Zürich, Stefano-Franscini-Platz 3, CH-8093 Zürich, Switzerland; svitas@ethz.ch (S.V.); jana@segmehl-energie.de (J.S.S.); iburgert@ethz.ch (I.B.)

[2] Applied Wood Materials, EMPA—Swiss Federal Laboratories for Materials Science and Technology, Überlandstrasse 129, CH-8600 Dübendorf, Switzerland

[*] Correspondence: cabanee@ethz.ch; Tel.: +41-44-633-75-58

Received: 07 December 2018; Accepted: 24 January 2019; Published: January 2019

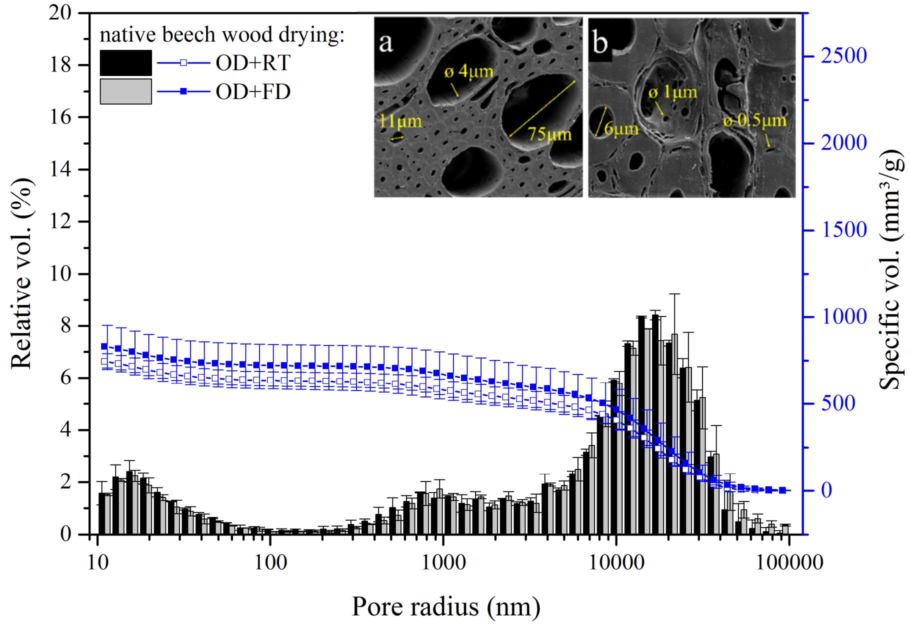

Figure S1. Cumulative pore volume and histogram of relative pore volume as a function of the pore radius of beech wood held under ambient conditions at room temperature after oven drying (OD + RT) or with an additional freeze-drying step (OD + FD) prior to measurement. Scanning electron microscope (SEM) imaging of the cross-section of native beech wood exhibiting various sized cavities in its structure in the range of the (**a**) upper and (**b**) lower level of the macropores.

The measurement of porosity requires dry samples; freeze-drying is one of the possibilities, especially when dealing with heat sensitive materials like wood. Air-dry samples were immersed in nitrogen and evacuated under vacuum in order to remove bound water. This additional drying should remove the water from humidity adsorbed onto the sample when stored under standard conditions (room temperature: RT). We conducted porosity measurements on native beech wood samples with this additional drying (OD + FD) and without (OD + RT). We did not observe any significant differences between samples subjected to an additional freeze-drying step or simply kept under ambient conditions. Based on this experiment, we did not perform this additional drying step for the oven drying treatment to conduct the porosity measurement.

Table S1. Pore sizes and pore size distribution of native beech wood from mercury intrusion porosimetry.

	Relative Pore Volume (%)	
**Pore size Range (nm)**	**OD + FD** [(a)]	**OD + RT** [(b)]
2'000–58'000	71.4 ± 3.23	70.73 ± 0.88
500–2'000	10.37 ± 0.95	10.33 ± 0.46
80–500	2.15 ± 0.25	3.02 ± 0.55
3.6–80	12.75 ± 0.93	14.29 ± 1.26

[(a)]Additional freeze-drying after oven drying; [(b)]Room temperature drying after oven drying.

Table S2. Pore sizes and pore size distribution from mercury intrusion porosimetry of delignified beech wood under harsh conditions.

	Relative Pore Volume (%)		
**Pore Size Range (nm)**	**OD + FD** [(a)]	**OD + RT** [(b)]	**FD** [(c)]
2'000–58'000	74.88 ± 2.69	69.09 ± 2.50	81.45 ± 0.43
500–2'000	9.07 ± 0.68	2.21 ± 0.20	5.75 ± 0.32
80–500	1.12 ± 1.12	0.30 ± 0.10	4.25 ± 0.44
3.6–80	0	1.74 ± 1.02	1.53 ± 0.34

[(a)]Additional freeze-drying after oven drying; [(b)]Room temperature drying after oven drying; [(c)]Freeze-drying as only drying.

Table S3. Pore sizes and pore size distribution from mercury intrusion porosimetry of delignified beech wood under mild conditions.

	Relative Pore Volume (%)	
**Pore size Range (nm)**	**OD + FD** [(a)]	**OD + RT** [(b)]
2'000–58'000	83.55 ± 0.72	82.75 ± 0.34
500–2'000	8.73 ± 0.29	7.08 ± 0.27
80–500	0.93 ± 0.03	0.77 ± 0.18
3.6–80	2.54 ± 0.96	2.64 ± 1.15

[(a)]Additional freeze-drying after oven drying; [(b)]Room temperature drying after oven drying.

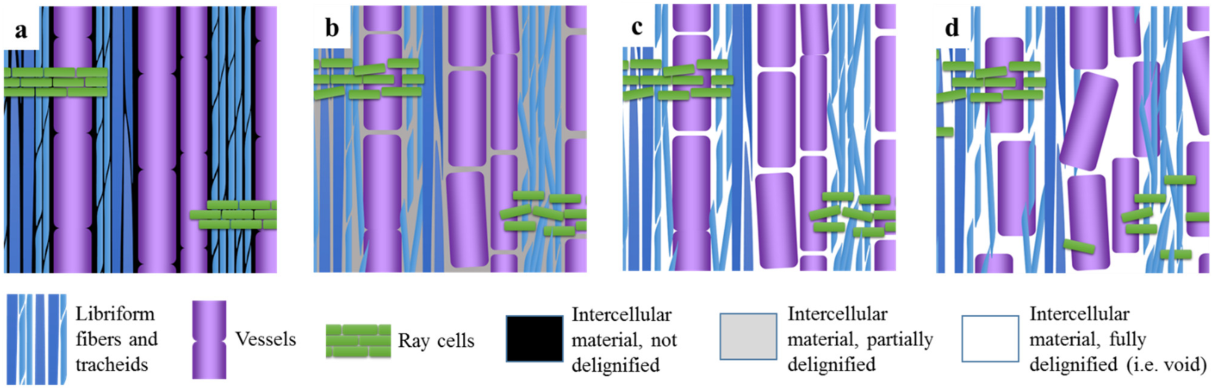

Figure S2. Schematic representation of wood tissue (**a**) and the disassembly of the cells (fibers, tracheids, vessels, ray cells) from the bulk (**b**–**d**) upon the weakening of the intercellular matrix through delignification.

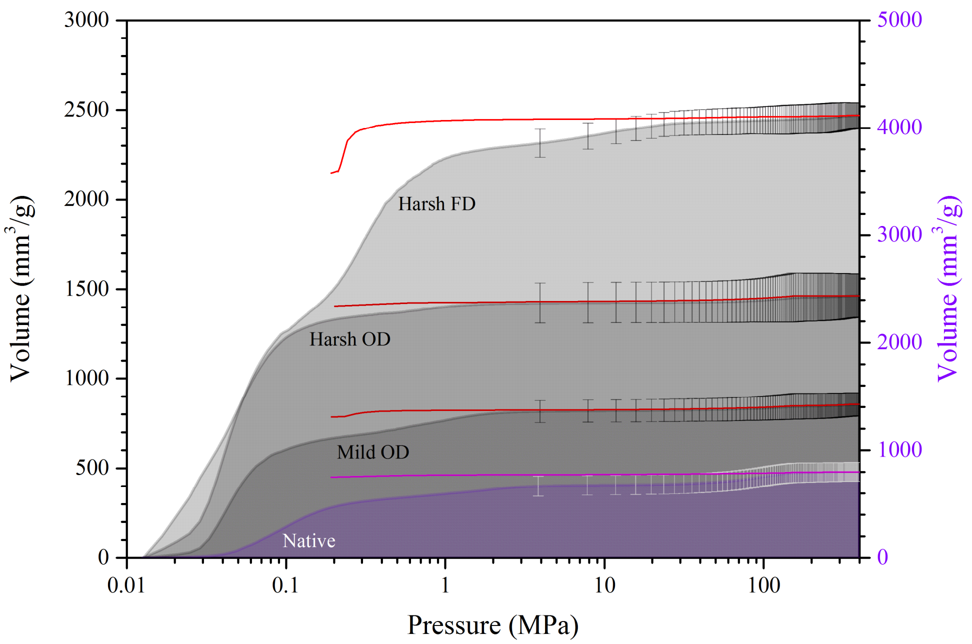

Figure S3. Volume-Pressure-Curves showing the intrusion curve (red/purple line) and the extrusion curves for raw (lilac) and delignified wood (grey) and the drying method (OD: oven dried, FD: freeze-dried).

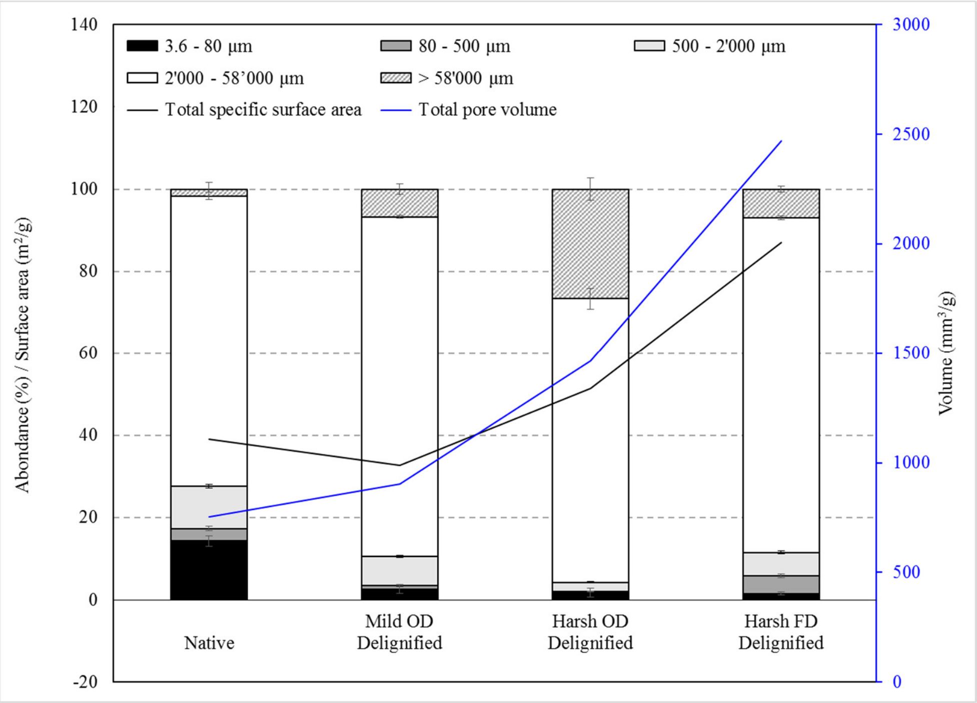

Figure 4. Overview of the MIP analysis (pore size distribution, total specific surface area and pore volume) of native and delignified beech wood under mild or harsh conditions, subjected to oven drying (OD) or freeze-drying (FD).

.

materials

MDPI

Table S4. Total pore volume and surface area as well as porosity determined by mercury intrusion porosimetry (MIP) and pycnometry for native and delignified beech wood.

	Porosity [1] [%]	Porosity [2] [%]	Total Pore Volume [1] [mm³/g]	Total Pore Surface Area [1] [m²/g]
Native	52.52 ± 2.85	54.429 ± 0.002	754.07 ± 39.64	38.97 ± 2.79
Delignified mild OD	54.07 ± 1.70	57.599 ± 0.001	901.74 ± 41.51	32.70 ± 4.14
Delignified harsh OD	65.70 ± 2.17	59.949 ± 0.001	1464.14 ± 85.24	51.34 ± 1.92
Delignified harsh FD	78.43 ± 1.56	78.974 ± 0.004	2470.07 ± 49.10	86.98 ± 1.55

[1] MIP measurement, [2] pycnometry, OD—oven drying, FD—freeze-drying.
